# Synthesis of Corn Starch Derivatives and Their Application in Yarn Sizing

**DOI:** 10.3390/polym12061251

**Published:** 2020-05-30

**Authors:** Stana Kovačević, Ivana Schwarz, Suzana Đorđević, Dragan Đorđević

**Affiliations:** 1 Department of Textile Design and Management, Faculty of Textile Technology, University of Zagreb Prilaz baruna Filipovića 28a, 10000 Zagreb, Croatia; stana.kovacevic@ttf.unizg.hr; 2 Textile Department, Higher Technological and Artistic Professional School Leskovac, Vilema Pusmana 17, 16000 Leskovac, Serbia; szn971@yahoo.com; 3 Textile Department, Faculty of Technology, University of Nis, Bulevar Oslobodjenja 124, 16000 Leskovac, Serbia; drdrag64@yahoo.com

**Keywords:** corn starch, sizing, cotton yarns, graft polymerization, rheological properties

## Abstract

The use of synthesized natural starches for the sizing process in fabric production is primarily an environmental contribution. Synthesized corn starch is environmentally friendly and productive, showing good results in cotton yarn sizing. Acrylamide (AA) and 2-hydroxyethyl methacrylate (HEMA) were applied for the grafting process of corn starch, and the initiators azobisisobutyronitrile (AIBN), potassium persulfate (KPS), and benzoyl peroxide (BP) were chosen to form the grafted monomers more effectively. The application of synthesized corn starch has been confirmed, especially with the AIBIN initiator in the grafting process of HEMA onto starch. The FTIR analysis confirmed that new and efficient products for sizing cotton yarns based on natural raw material (corn) were developed. The research showed that the synthesized corn starch improved physical-mechanical yarn properties and abrasion resistance and reduced yarn surface hairiness. Ultrasonic desizing of yarn and the use of a lower size concentration led to better results than desizing by washing, and the Tegewa numbers confirmed that the desizing process was successful.

## 1. Introduction

Sizing is one of the most complex, but also one of the most expensive steps in fabric production, unfortunately still one of the biggest wastewater polluters in the textile industry. Cotton warp yarn sizing cannot usually be avoided if the quality and economic production of fabrics is conditional. Sizing increases warp yarn strength, abrasion resistance, and reduces surface hairiness and static electricity, all of which are crucial parameters for achieving necessary conditions in fabric production. When warp yarn sizing was introduced, natural starches, such as corn, potato, wheat, etc. were applied. Their disadvantage was the size of molecules, which hardly penetrated the interstices of the threads, viscosity instability when changing the temperature, formation of a rigid film on the thread surface, decrease in yarn elasticity, rapid rotting, gel appearance, formation of foam in the size box, uneven size pick up, size removal in the weaving process, higher number of breaks, desizing with microorganisms, and inability to recycle [1,2,3].

The modification of natural starches reduces or even eliminates all deficiencies of natural starches. Starch modification reduces retrogradation, paste gelling tendencies and gel syneresis, improves paste clarity and gloss, as well as paste and gel texture. In this way, starch adhesion is improved, and a protective film is formed around the thread, which is extremely important for hydrophobic synthetic fibres [4]. The modification of starch has triggered a revolution in new technological processes and market trends. These highly functional derivatives should create a competitive advantage for the new product. There are four basic types of modification, namely chemical, physical, enzymatic, and genetic [5,6,7,8,9,10,11,12]. 

Chemical modification of starch includes polymeric molecules of starch granules in their native form. Modification is achieved by derivatization such as etherification, esterification, and thus crosslinking, oxidation, catonization, and starch grafting. There is a trend of combining different types of chemical treatments to create new types of modifications [13,14,15,16,17,18,19,20]. Similarly, chemical methods have been combined with physical modifications such as microwaves, radiation and extrusion to produce modified starch with specific functional properties that can be implemented not only in the sizing process, but also in the production of textile composites with exceptional properties, while being ecologically and economically justified [21,22,23,24,25,26,27,28].

With the use of chemical agents, the quality of the fabric and the cost-effectiveness of production increased significantly, while the negatives of synthetic agents remained in the background, such as: environmental pollution, danger to human health, aggressiveness of the agents significantly affects the respiratory tracts of the workers who are daily present during the sizing process, destroying parts in the size box such as rollers, bearings, measuring devices and box walls. Despite this fact, the textile industry applies chemical sizing agents and has almost completely replaced natural modified agents. However, improvements in the sizing process are now reflected in the development and application of new sizing agents, all with the aim of achieving the most economical, efficient and environmentally friendly product [29,30].

Modification of natural starches reduces or almost eliminates all deficiencies of natural starches. As environmentally friendly products, they should gradually replace fully synthetic sizing agents such as polyvinyl alcohol (PVA), carboxyl methyl cellulose (CMC), polyacrylate (PA), and other synthetic agents used in the sizing process today [31,32,33].

The selection of sizing agents, the conditions and methods of sizing, and the optimization of size pick-up on the yarn constitute one of the greatest challenges for technologists in the sizing and weaving process. It is often very difficult and almost impossible to determine the appropriate recipe for each type of yarn due to the deviation of the properties of the yarn, the types and proportions of sizing agents, the sizing and weaving conditions.

**Figure 1:**
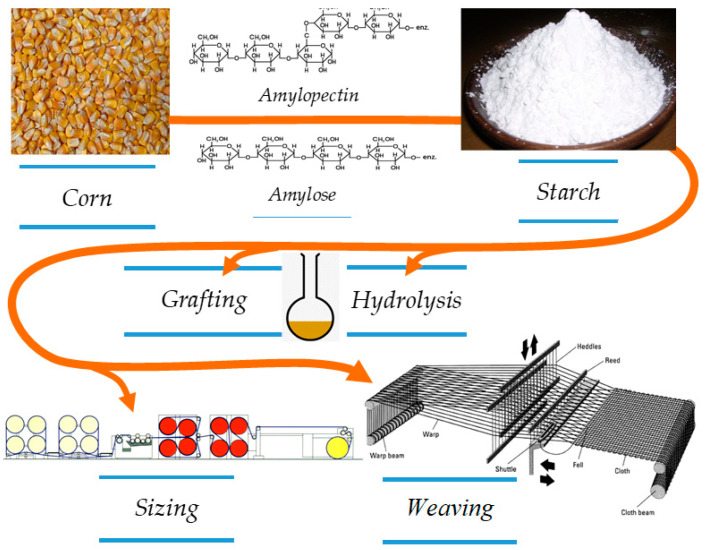
Preparation procedure of hydrolysed and grafted corn starch, sizing and weaving process.

The synthesis of modified starches and the verification of their efficacy itself is a type of activity that requires knowledge of the chemical and mechanical properties of the chemicals from which new active substances are synthesised in particular, as well as the textile materials to which these active substances are applied in order to fulfil their function under certain conditions in the weaving process (Figure 1).

**Figure 2:**
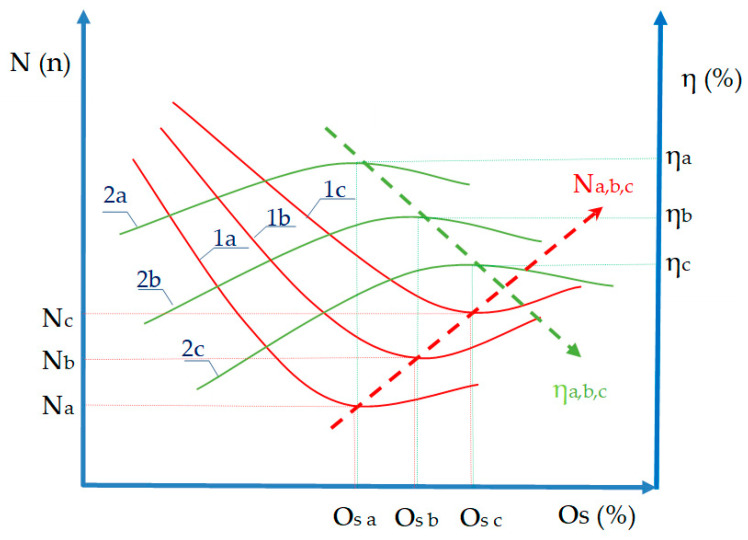
Size pick-up (Os/%), number of breaks on the loom in unit of time (N/n), loom efficiency rate (η/%); a,b,c—three yarn samples, ηa,b,c—maximum loom efficiency rate (%), Na,b,c—minimum number of yarn breaks on the loom (n), Osa,b,c—optimal size pick-up (%), 1a,b,c—curves indicating the relation between the number of breaks and size pick-up, 2a,b,c—curves indicating the relation between the machine efficiency and size pick-up, Na,b,c—course of increasing optimal size pick-up in the deteriorating yarn, ηa,b,c—course of reducing the loom efficiency rate in the deteriorating yarn.

As the size pick-up on the yarn increases, the number of breaks decreases significantly up to a certain limit when it starts to rise again (Figure 2), indicated by red curves. Optimal size pick-up is a point on the curves where there is a minimum number of breakages and maximum loom efficiency rate. The higher the optimal size pick-up, the greater the number of breaks (direction Na,b,c), and the loom efficiency rate decreases (direction ηa,b,c). The goal of sizing is actually to reduce the number of warp thread breaks in the weaving process, thereby increasing loom productivity, leading to a higher quality and more economic fabric [34,35]. Comprehensive research on the sizing process requires a broader knowledge of textile raw materials and technology, chemistry, ecology, the development and application of automation, which enables the monitoring and control of many parameters in the sizing and weaving process. This points to the need for teamwork and research in the development and application of new, more environmentally friendly, cheaper, and higher quality sizing agents. This research will be based on synthesizing corn starch as a possible substitute for synthetic sizing agents. A gradual replacement of synthetic by natural and environmentally friendly starch substitutes is only possible on the basis of scientific studies providing justified reasons.

## 2. Experimental

### 2.1. Materials

The investigation was carried out on cotton yarns with counts of 20 tex, 30 tex, 20 × 2 tex and 30 × 2 tex. 

For the purposes of this research the following active products were employed.: corn starch (25 mas.% amylose, “Jabuka” Starch Industry, Pančevo, Serbia), hydrochloric acid—HCl (Centrohem, Stara Pazova, Serbia), ethyl alcohol (ethanol)—C_2_H_5_OH (Reahem, Novi Sad, Serbia), and sodium carbonate—Na_2_CO_3_ (LG Hemija, Belgrade, Serbia). The following monomers were employed:

- acrylamide (AA) C_3_H_5_NO (Merck KG-aA, Darmstadt, Germany),
- 2-hydroxy-ethyl methacrylate (HEMA) C_6_H_10_O_3_ (Sigma-Aldrich, St. Louis, MO, USA).

The following initiators were used in the grafting process:

- azobisisobutyronitrile (AIBN) i.e., (2,2′-azobis (2-methylpropionitrile)) 99% (Sigma-Aldrich, St. Louis, MO, USA),
- potassium persulfate (KPS) K_2_S_2_O_8_ (Centrohem, Stara Pazova, Serbia),
- benzoyl peroxide (BP) (C_6_H_5_CO)_2_O_2_ (Sigma-Aldrich, St. Louis, MO, USA) [36].

### 2.2. Methods and Testing Machines

The yarn sizing process was carried out on an innovative laboratory sizing machine adapted to industrial conditions, University of Zagreb Faculty of Textile Technology, Zagreb, Croatia [36]. Microscopy with magnification 10× and 40× was performed on a Microscope Ceti, Belgium. Breaking force and elongation at break of the yarn was tested on a Textechno tensile tester, model Statimat M (Textechno H. Stein GmbH & Co. KG, Moenchengladbach, Germany), according to standard ISO 2062. The FTIR analysis of samples-potassium bromide technique, spectrophotometer BOMEM Hartmann & Braun MB-Series (ABB group, Zurich, Switzerland) in the range of wavelengths 4000–400 cm^−1^ was conducted. Yarn hairiness was tested on a Zweigle G 565 instrument (Zweigle, Reutlingen, Germany) for yarn hairiness testing according to standard ASTM D 5674-01. Abrasion resistance of the yarn was measured on the Zweigle G551 yarn abrasion tester (Zweigle, Reutlingen, Germany). 

The HPLC method (high pressure liquid chromatography) was implemented to examine the residual amounts of unreacted monomer in the copolymer. Tests were carried out on the device Agilent Series 1100 HPLC (Agilent Technologies, Santa Clara, USA) with diode-array detector, DAD 1200 Series. The detection wavelength was 205 nm. The ZORBAX Eclipse XDB-C18 column (Agilent Technologies, Santa Clara, USA), 4.6 × 250 mm, 5 μm was used. The effluent was methanol, the flow rate was 1 mL/min, the column was thermostated at 25 °C and the injected volume was 20 μL. Monomer standards were prepared by single weighing of the sample with diluted methanol.

Apparent viscosity was determined on the rotational viscometer “Visco Basic Plus” (Fungilab S.A., Barcelona, Spain). Starch solutions were prepared in a way that the starch was dispersed in distilled water, after which the solution was heated to 100 °C, with stirring at a speed of 150 rpm. When measuring viscosity, a spindle marked R3 was used, with a spindle speed of 60 rpm. Measurements were performed at different temperatures: 40 °C (reaching time 15 min), 60 °C (reaching time 20 min), 85 °C (reaching time 30 min).

Mean molar masses and molar mass distribution (*M*n, *M*w, *M*z, D) were determined using a GPC Agilent 1100 Series gel permeation chromatograph (Agilent Technologies, Santa Clara, USA) using a 1200 Series (Agilent Technologies, Santa Clara, USA) differential refractometer (RID detector) as a detector. A Zorbax PSM 300 column, 250 × 6.2 mm, 5 μm with a nominal molar mass range of 3 × 10^3^–3 × 10^5^ g/mol was used. In order to construct a calibration diagram for the purpose of quantitative processing of gel-chromatograms of the tested hydrolysed starch copolymers, calibration of Zorbax PSM-300 gel column was performed using dextran samples (Pharmacosmos, Holbaek, Denmark) with narrow molar mass distributions. Dextran standards, of known molar masses and the same concentrations as for the samples, were applied in the same way as the samples. Redistilled water was used as eluent, at a flow rate of 1 mL/min. The column was thermostated at 25 °C and the injected volume of the sample solution was 20 μL. Solutions of copolymers in redistilled water at a concentration of 4.5 mg/mL were used for analysis, which were filtered through a 0.45 μm filter before measurement.

The size pick-up (D) on the yarn was determined by mass technology. During the sizing process, percentages of the copolymer solution in the water (concentration) for the single yarn were 5%, 10%, 15%, and for the ply yarn: 1%, 3%, and 5%, adapted to industrial conditions.

Desizing of sized yarn samples was performed using a non-ionic detergent, Lavan NH (Textilcolor AG, Sevelen, Switzerland), concentration 2 g/L, with a ratio of 1:30 in the Ahiba Linites apparatus. The aqueous solution of this agent was heated at 60 °C for 5 min, before a sized yarn sample was added and treated for 30 min at the same temperature. Finally, the sample was washed abundantly with water and air-dried. Furthermore, one part of the experimental desizing was carried out applying ultrasonic energy with an ultrasonic bath (Sonic, Niš, Serbia). The frequency of applied ultrasonic oscillations was 40 kHz, power used was 150 W, time 30 min, and temperature 60 °C. The degree of desizing (%) was obtained by gravimetric calculation based on mass differences:

$$D_{d}=\frac{m_{bd}-m_{ad}}{m_{bd}}\cdot 100$$

where: m_bd_ denotes mass before desizing (g), and m_ad_ mass after desizing (g).

To prepare the samples before measurement, they were conditioned in a thermal chamber with the following conditions: temperature 20 ± 1 °C, humidity 65 ± 2%.

The Tegewa method was used to identify residual starch derivatives with the aid of iodine solution. The iodinated solution was prepared by dissolving 10 g of potassium iodide in 100 mL of water, followed by the addition of 0.65 g of iodine and dilution with water to an amount of 800 cm^3^. Finally, the solution was supplemented with ethanol to increase the content to 1 L. The raw material was immersed in this solution and kept for 1 min, after which it was washed in cold water. This was followed by the absorption of excess water with the filter paper and a quick comparison with the Tegewa scale [37].

### 2.3. Graft Copolymer Synthesis Process

The hydrolysis process [38,39] was carried out as follows: to a solution of 1 N hydrochloric acid and water at a temperature of 60 °C starch was added. After the expiry of the reaction time, the reaction product was precipitated in 100 mL of ethyl alcohol and neutralized with a dilute 1% sodium carbonate solution, washed and finally dried in an electric oven at 60 °C for 3 h. The whole process was carried out with intensive stirring on a magnetic stirrer. The resulting sample was designated as HS.

The grafting process with the initiators AIBN, KPS, BP [40,41,42] was carried out as follows: an aqueous solution of 10 g of hydrolysed starch was heated for 15 min at 50 °C in a water bath, followed by the addition of 5 g of monomer (acrylamide or 2-hydroxy-ethyl methacrylate) and 1% of initiator (relative to starch weight + monomer). The temperature was kept at 50 °C for 120 min with reflux and intensive stirring on a magnetic stirrer.

The reaction product (hydrolysed starch grafted with AA) was precipitated in 100 mL of ethanol. After straining, the precipitate was washed for 10 min with ethanol at room temperature and then with ethanol-water solution in a ratio of 80:20, 3–5 times [43]. Finally, the precipitate was dried in an electric dryer at 60 °C. The removal of the unreacted monomer and homopolymer from the product obtained by grafting 2-hydroxy-ethyl methacrylate onto a hydrolysed staple was performed in a Sokhlet apparatus using methanol [44,45,46].

Table 1 lists the designations of the copolymers obtained after graft copolymerization onto starch. When copolymerizing with AIBN, KPS, and BP initiators, the grafted branches are bonded with the starch skeleton via oxygen, because graft copolymerization is initiated by the release of active hydrogen from the starch hydroxyl [47].

## 3. Results and Discussion

### 3.1. Characterization of HS-AA and HS-HEMA Copolymers

Table 2 presents the individual parameters showing the hydrolysis efficiency and the performance of grafting monomers onto corn starch, depending on the initiator type. Copolymerization by starch grafting with vinyl monomers was accompanied by the homopolymerization of monomers as reactants. Homopolymerization is a side reaction of copolymerization. If the activated chain, which advances and initiates homopolymerization, meets active copolymerization centres, the original homopolymerization is converted into copolymerization. Therefore, some of the homopolymers will become grafted homopolymers and the grafting efficacy may increase. Accordingly, the grafting ratio will also increase with increasing efficacy. As a result, increased grafting efficacy increases product efficiency, and with a similar grafting ratio, an increase in grafting efficacy reduces the amount of monomers required, which lowers production costs. Given the results of yield, percentage, and grafting efficacy, the BP initiator proved to be very successful in grafting AA onto starch, and the AIBN initiator in grafting HEMA onto starch. The yield of AA and HEMA grafting onto corn starch ranged from 79.33% to 84.67%, the grafting percentage ranged from 19 to 27%, the grafting efficacy percentage ranged from 38.31% to 54.88%, while the conversion of monomers into polymer ranged from 98.2% to 99.2%.

**Figure 3:**
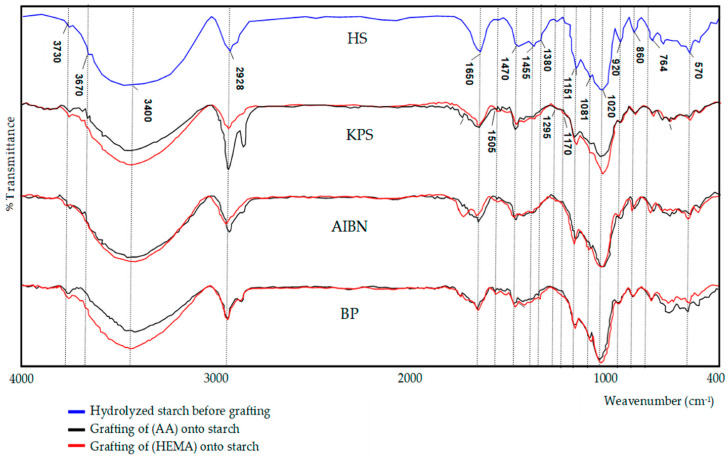
FTIR spectra of the hydrolysed and grafted starch per individual types.

Figure 3 shows the FTIR spectra of hydrolysed and grafted corn starch, where comparative peaks per starch initiators are visible. A broad peak of 3400 cm^−1^ is observed, which stems from O–H valence vibrations as well as a smaller peak at 2925 cm^−1^, which is attributable to the C–H valence vibration. Wave numbers at about 1153, 1078 and 1023 cm^−1^ describe C–O–C stretching (triplet of starch) [48]. In the case of starch grafted with acrylamide, the OH valence band of the starch hydroxyl group and the NH valence band of the poly (acrylamide) amide group overlap and lead to a peak at 3730 cm^−1^ resting on a peak occurring at about 3670 cm^−1^. Peaks at about 570, 764, 860, and 920 cm^−1^ (vibrations of the –OH group) in grafted starch change in intensity and shape, indicating that OH starch groups changed during the reaction. Thus, the presence of these additional peaks in the case of grafted starch as compared to hydrolysed starch confirms the successful grafting of poly (acrylamide) chains onto starch [40]. The FTIR spectrum of starch with HEMA grafting initiator shows the following bands of the monomer: valence vibrations of C–O at 1295 and 1170 cm^−1^, valence vibrations of C=C double bonds at 1650 cm^−1^, vibrations of C–H bond; νs (CH3) at 2928 cm^−1^, and the valence vibrations of the OH group at about 3420 cm^−1^. Vibrations of the ring of the aromatic core were observed at 1600, 1505, and 1470 cm^−1^. Asymmetric and symmetric stretching vibrations of the methyl group were observed at 1455 and 1380 cm^−1^ [49].

The amounts of residual AA and HEMA monomer in the copolymer samples after grafting onto hydrolysed starch are shown in Table 3. The maximum amount of the monomer was retained in the sample HS-AA-KPS (6.14 mg/g), followed by the sample HS-HEMA-BP (5.75 mg/g), and the smallest amount in the sample HS-AA-BP and HS-HEMA-AIBN (4.46 mg/g), confirming a more effective graft polymerization in the presence of these initiators.

After chromatography of a suitable sample of starch copolymer, depending on the detector used (RID), ChemStation software provides an appropriate chromatogram with characteristic chromatographic parameters. The program offers the possibility to activate the GPC software and transfer the obtained data to process the chromatograms and analyse the molecular masses of each separated fraction of the analysed polymer. 

About 0.2 g of powdered, dried copolymer was used for analysis. The sample was extracted with 10 mL of methanol at room temperature for 48 h with occasional stirring. The extracts were filtered through a 0.45 μm filter and used for HPLC analysis.

**Figure 4:**
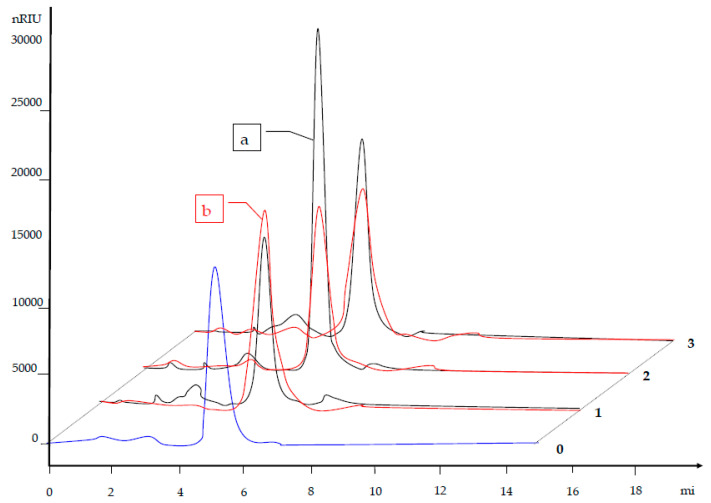
HPLC chromatograms; a—AA starch copolymer, b—HEMA starch copolymer; 0—hydrolysed starch, 1—grafted with AIBIN initiators, 2—grafted with BP initiators, 3—grafted with KPS initiators.

Figure 4 and Table 4 show the HPLC chromatograms (RID signal) of aqueous extract of the synthesized starch copolymer samples (AA and HEMA), depending on the type of initiators used. It is evident that under the given conditions, depending on the initiators used, an appropriate response of the chromatographic signal of the copolymer occurs. It is noticeable that on the chromatogram of the AA monomer grafted with the BP initiator the peak height is the highest and the largest area is below the diagram.

**Figure 5:**
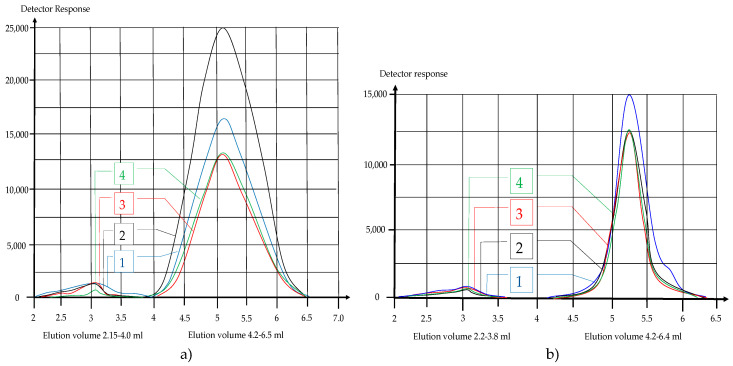
Signal on the RID detector (GPC data) depending on elution volume, (**a**) AA starch copolymer; (**b**) HEMA starch copolymer, 1—HS-AA-AIBN, 2—HS-AA-BP, 3—HS-AA-KPS, 4—HS.

GPC software was used to process the chromatograms of the starch copolymer samples. Figure 5 shows the eluates relating to the second significant peak at about 5.2 mL of the elution volume, with an intensity of 10 or more times in relation to the signal occurring in the previous case. This may indicate that this area (about 5.2 mL) contains the largest number of macromolecules of the approximate molar mass of the grafted product.

### 3.2. Molar Masses

The results obtained are consistent with the well-known fact that molecules of natural polymers, e.g., polysaccharides, and practically all synthetic polymers differ in size. These polymers are non-uniform or polydisperse, meaning that molecules of different sizes with different proportions can be found in the material sample. For example, it may be the case that two polymer samples with the same average molar mass have completely different properties because in one sample all molecules are approximately of the same size and in the other there are many relatively small or large molecules. Since all tested samples have *M*w > *M*n (especially expressed in higher molar masses), i.e., they are polydisperse systems, the rule applies: the higher the difference between *M*w and *M*n, the more polydisperse the system is (Table 5).

All synthetic polymers are known to be polydisperse, so the order *M*z > *M*w > *M*n applies to them [50]. An increase in the polydispersity index (example HS-AA-AIBN) means there is a higher proportion of chains with lower molar mass values, whose mobility is therefore higher. The polydispersity index, which reflects the circumference of the macromolecule with its high circumferential values, assumes a product that has a wide distribution and can contain large particles or aggregates.

Greater chain lengths, i.e., greater molar masses, ensure a greater number of interactions and the possibility of greater interlacements of the flexible chains in the yarn, giving them greater strength and elasticity. The fluidity of the solution of such materials decreases dramatically when the chains become very long, making it difficult to form a uniform surface film on the yarn. Therefore, compromise values of these parameters are required to optimize the given set of properties. The deviation of the polydispersity index provides additional freedom in the design of polymer materials. Higher polydispersity ensures better processability. One explanation for this phenomenon is that shorter chains are more easily movable in the solution and serve as “lubricants” (plasticizers) when longer chains or their aggregates are moved [51]. Table 5 shows the different types of molar masses of the copolymer samples as well as the degree of polydispersity calculated from the distribution curve. The samples of hydrolysed graft starch or lowest Mw with an elution volume of 2.15–4.0 mL show the highest homogeneity. Smaller molar masses of the grafted samples with an elution volume of 4.2–6.5 mL result in poorer homogeneity.

### 3.3. Microscopy of the Copolymer Solution

**Figure 6:**
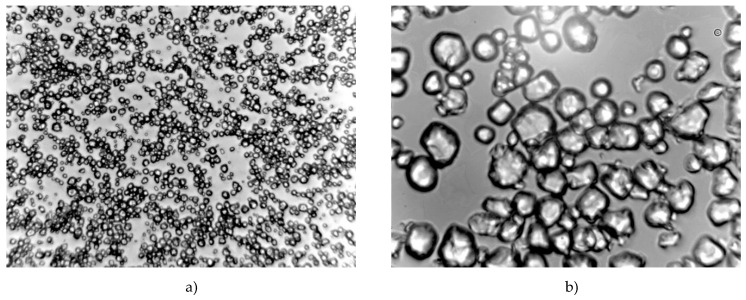
Microscopic appearance of the solution of HS-AA-10 copolymer; regime: 40 °C—10 min (**a**) magnification: 10×; (**b**) magnification 40×.

**Figure 7:**
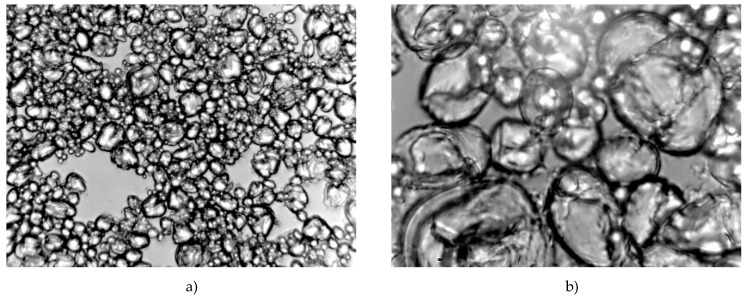
Microscopic appearance of the solution of HS-AA-10 copolymer; regime: 40 °C—60 min (**a**) magnification: 10×; (**b**) magnification 40×.

Microscopic representation of starch copolymer solution can help to understand the rheological behaviour of macromolecular chains in the solution. Corn starch granules have an oval and spherical shape, some of the granules have an irregular shape with individual sharp edges the size of which ranges from 2–30 μm (usually 10–15 μm). The layers are not visible and show a three-pointed crack or a bright cavity in the middle. Small grains (2–8 μm) are round and interconnected [14]. There is no obvious structural difference between acid hydrolysed and modified starch (Figure 6 and Figure 7). With a longer dormant state of the size changes occurring on the surface of acid hydrolysed starch granules, the surface of the granules becomes less smooth, and the size increased.

Starch granules become thinner and more easily attacked by hydrogen ions, which agrees with the fact that amorphous regions of starch granules are primarily degraded by acid hydrolysis. Since the amorphous regions are mainly located within the starch granules and the crystalline regions mainly in the outer part of the starch granules, the starch granules become thinner during the first acid hydrolysis phase and break up at a later stage. Figure 6 and Figure 7 show microscopic images of an HS-AA-10 starch copolymer solution after dissolving at 40 °C and a 10–60 min dormant state. Changes are obvious as starch granules are associated and begin to swell.

### 3.4. Viscosity

Starch properties depend on the contents of amylose and amylopectin, but also on other components contained in the starch granule, such as phosphates, lipids, phospholipids, etc. However, amylopectin, as a basic starch component, has a dominant effect on starch properties [52,53]. The length of the amylopectin side chains affects the clustering, retrogradation, and starch properties of the solution.

During the disintegration of starch granules, the release of amorphous amylose first occurs, building a three-dimensional network beyond starch granules and inhibits further swelling of starch granules. In addition to amylose, the present lipids also inhibit swelling. Although amylose contributes only slightly to the viscosity of the starch solution after granule disintegration, the viscosity increases with increasing amylose content after cooling of the starch solution, which is explained by the crystallization of amylose and the formation of a gel structure.

It is known that starch solution is retrograded during the starch dormant state, which is one of the reasons for the poor performance. During starch retrogradation, external short side chains of amylopectin are rapidly transformed from the ball into a helix structure by associating the external short chains and by forming a B-type crystalline structure, whereas amylose forms double helix associates. Starch retrogradation is also influenced by the presence of phosphates, lipids and phospholipids, temperature, solution concentration, pH of solution and the presence of some ingredients. Retrogradation accelerates with increasing concentration and decreasing temperature of the solution. Very short chains (degree of polymerization of 6–9) suppress starch retrogradation [54].

For the purpose of this research, starch derivatives were prepared in concentrations of 1%, 3%, 5%, 10%, and 15%. During the preparation of the solution, it was observed that the solution was opalescent and that at lower concentrations up to 3%, the hydrolysed corn starch solution acted almost like a Newtonian fluid.

This means that starch macromolecules are sufficiently distant from each other to prevent strong physical cross-linking, and that finally formed bonds are easily broken by mechanical forces and fail to be formed again in a relatively short time in the tested range of oscillation frequencies. It is known that the solubility of natural starch increases significantly with hydrolysis, i.e., polar vinyl monomer copolymerization [55].

Viscosity as an indicator of the size of starch molecules assumes that their higher values imply larger macromolecules and subsequently lower solubility and higher viscosity (e.g., HS-HEMA). The high grafting content will now disrupt the intermolecular bonds of the starch chains and increase hydrophobicity. In the constellation of relations between hydrophilic and hydrophobic interactions, the influence of secondary chemical bonds, grafted macromolecules show specific behaviour in solution, which is reflected in the viscosity and also in the binding to cotton yarn in its impregnation processes. It is noticeable that the viscosity of all solutions of copolymers of HEMA monomers has a slightly higher value, which implies the assumption that grafting with this monomer partially reduces the solubility of hydrolysed starch. 

Taking into account that the same concentrations of hydrolysed starch solution had a slightly lower viscosity, it can be argued that starch grafting with vinyl monomers increased the viscosity of the solution, i.e., the size of the hydrolysed starch molecules increases during grafting. The presence of grafted chains in the molecular structure of starch causes significant changes in the behaviour of starch in aqueous solutions. In addition to loosening the starch structure, grafted chains appear to be able to associate molecules. Finally, the chains grafted onto the hydrolysed starch increase the molar mass and lead to possible cross-linking, since it is possible to create different types of secondary chemical bonds (aqueous, dipole, etc.). It is noticeable that, with decreasing starch concentration, the viscosity declines as expected (Table 6). The influence of the temperature of the copolymer solution on the viscosity starting at 40 °C is evident, first rising to a temperature of 60 °C, and finally decreasing at a higher temperature of 85 °C, at the same concentration. Namely, by increasing the temperature, the grafted starch granules begin to disintegrate, the entire structure of the starch macromolecules begins to weaken, i.e., after irreversible swelling, dissolution finally occurs, which increases the viscosity at the temperature of the gelling point (60–70 °C). Finally, by further increasing the temperature, complete disintegration and a slight decrease in viscosity occurs. Minor differences in viscosity confirm that the copolymer has shorter chains and that it is more soluble than hydrolysed starch. All grafted products with corresponding designations or concentrations increase the viscosity compared to the non-grafted hydrolysed product, which is an indication of the good stability of potential starches. The differences depend on the temperature at which the viscosity is measured, but generally they are greater by several units mPa·s.

For example, the hydrolysed starch designated as HS-5 at 40 °C had a viscosity of 38.8 mPa·s, while the highest value for the same concentration was observed for the grafted hydrolysed starch designated as HS-HEMA-5, which at 40 °C had a viscosity of 44.8 mPa·s; it is also similar at other temperatures, so HS-5 had a viscosity of 57.1 mPa·s at 60 °C, while the highest value for the same concentration was observed for the grafted hydrolysed starch designated as HS-HEMA-5, which had a viscosity of 62.0 mPa·s at the same temperature. By increasing the viscosity, the surface size pick-up on the yarn is higher in contrast to the low viscosity, which at the same time assumes a lower size pick-up on the surface of the yarn, but probably more uniform and larger in the interior, which is an additional necessary requirement in the sizing process. As a rule, higher size temperature means lower viscosity, which is not the case with the applied starch, but it is certain that higher temperature allows a faster movement of size molecules, and thus a higher degree of overcoming adhesion forces and a better adsorption of the size to the fibres in the yarn. 

Considering the mentioned effects, it can be claimed that the effective yarn sizing process depends on: concentration, viscosity, size temperature, recipe, size dormant state, and on the sizing conditions such as: yarn tension, sizing speed, the pressure of the squeeze rollers, drying method and drying temperature, etc. The current mutual influence of these parameters, antagonism or synergism, will determine sizing efficiency and quality. 

### 3.5. Size Pick-up

**Figure 8:**
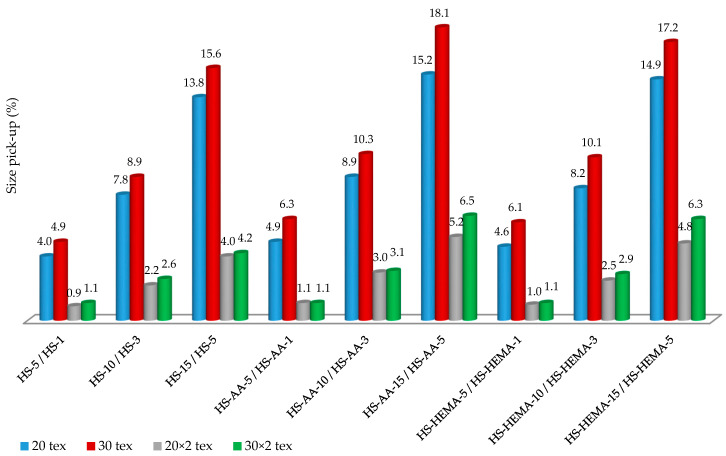
Size pick-up on the yarn sized with hydrolysed starch, then with the starch grafted with AA and HEMA monomers; explanation of designations: “HS-5/HS-1“: HS—hydrolysed starch, 5—concentration (5%), 1—concentration (1%).

By increasing the size concentration (for single yarn: 5%, 10%, and 15%; for ply yarn: 1%, 3%, and 5%) the size pick-up on the yarn increases in all samples (Figure 8). Hydrolysed starches have a lower size pick-up than the grafted starches at the same concentration. It can be claimed that the HS-AA starch shows a higher size pick-up than HS-HEMA starch. Comparing the size pick-up between the single and ply yarn, the single yarn had a higher size pick-up, which can be attributed to a looser structure (greater yarn porosity) [56].

### 3.6. Breaking Strength and Elongation at Break

**Figure 9:**
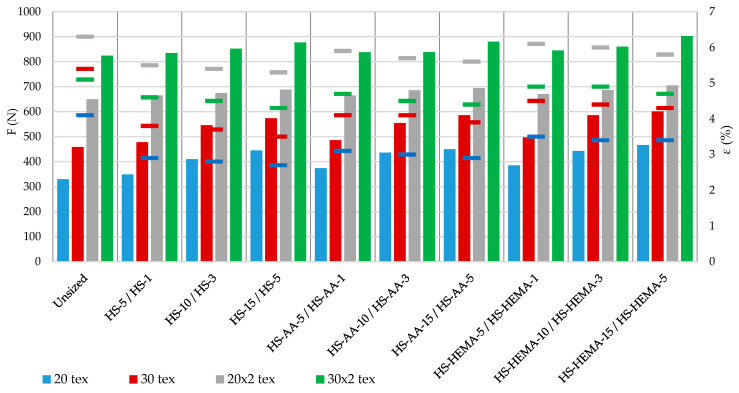
Breaking forces and elongation at break of the yarn.

Starch hydrolysis caused an increase in the breaking forces in all samples. Starch grafting caused an additional increase in breaking forces. The HEMA grafted starch achieves better results than AA in terms of breaking forces for all yarn types, which indicates its correct arrangement and good adhesion both on the surface and inside the yarn. Due to a higher concentration, the breaking force was higher for the single yarn (concentrations 5%, 10%, and 15%) and the plied yarn (concentrations 1%, 3%, and 5%), but elongation at break mostly decreased (Table 7, Figure 9).

### 3.7. Yarn Abrasion

**Figure 10:**
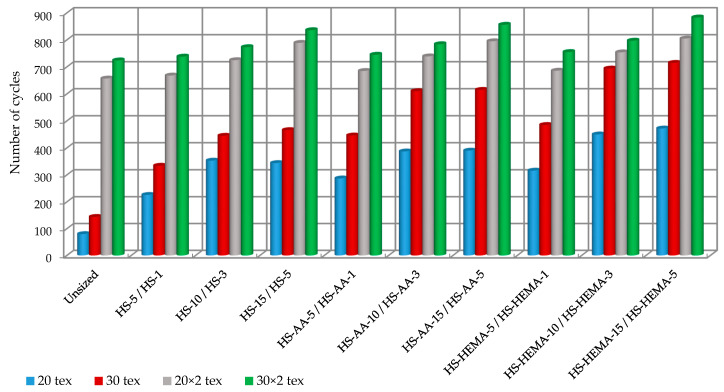
Yarn abrasion resistance during the sizing process.

One of the aims of yarn sizing is to increase the abrasion resistance of the yarn so that the yarn running through the elements of the weaving machine (drop wires, heald shafts, and reed) can withstand without breaking [57], as shown in Figure 10.

Grafted starch proved to be an efficient product with regard to abrasion resistance, especially HS-HEMA, which can be sought in its hydrophobicity. The waterproof behaviour or more difficult dissolution of HS-HEMA starch decreases if more monomers are grafted on it. High grafting content disrupts the intermolecular bonds of starch chains and increases hydrophobicity. In the constellation of relations between hydrophilic and hydrophobic interactions, the influence of secondary chemical bonds, grafted macromolecules show specific behaviour in solution, which is reflected in viscosity and then in binding to cotton yarn in impregnation processes. It is noticeable that the viscosity of the copolymer solution of the HEMA monomer had a slightly higher value, which implies the assumption that grafting with this monomer partially reduces the solubility of hydrolysed starch. This resulted in a better yarn surface protection.

### 3.8. Yarn Hairiness

**Figure 11:**
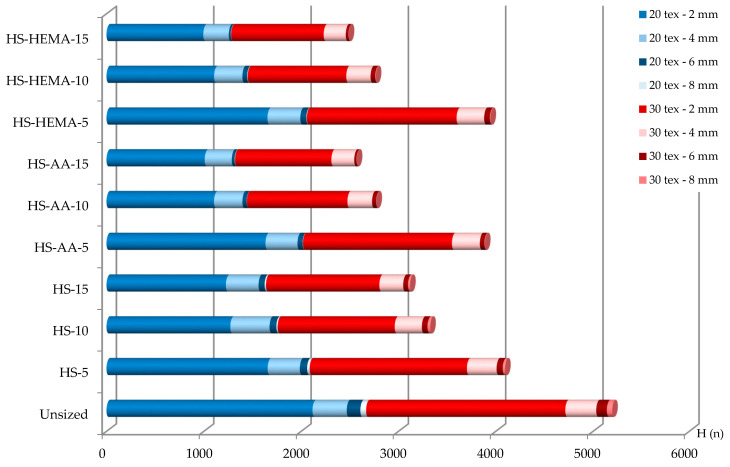
Hairiness of the single yarn in the sizing process.

**Figure 12:**
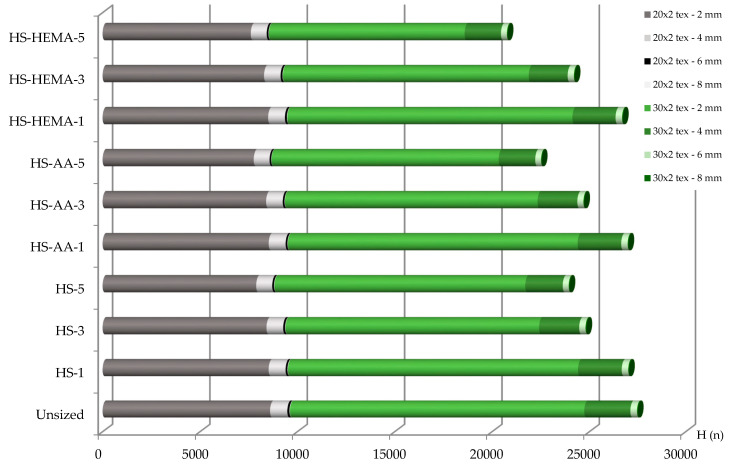
Hairiness of the plied yarn in the sizing process.

One of the goals of sizing is to reduce yarn hairiness, which has negative effects in the sizing and weaving process. High yarn hairiness causes mutual stickiness, higher yarn unevenness, higher abrasion and a greater number of yarn breaks on the loom [57]. Shorter fibres, being most numerous (up to 2 mm), do not cause major problems; however, if protruding fibres are longer, the problem becomes much worse. The hydrolysis of corn starch with subsequent grafting significantly reduced the yarn hairiness of all yarns (Figure 11 and Figure 12, Table 8). A higher size concentration also reduced yarn hairiness.

### 3.9. Degree of Desizing

Desizing was carried out in two ways: by using ultrasound and washing off (Table 8). Hydrolysed starch showed slightly lower desizing results compared to grafted starch. Better desizing results were observed in yarns with lower concentration, i.e., with lower size pick-up. Ultrasound proved to be more effective for desizing, namely for most hydrolysed and grafted starches [58,59].

Table 9 shows the results of the Tegewa classification of desizing performance. The values of Tegewa numbers ranged from 5 to 8. The recommendation is that Tegewa numbers from six upwards are quite sufficient to evaluate the desizing process as “successful”. For all grafted starches, Tegewa numbers six and above were predominant, meaning that the desizing process was successful [60]. Desizing by ultrasound gave better results for all samples than desizing by washing. 

## 4. Conclusions

The following conclusions can be drawn from this research:

- The modification of corn starch was carried out with the aim of producing an environmentally friendly and effective sizing agent that works better than starch.
- Acrylic monomers were grafted onto the starch structure using the initiators AIBN, BP, and KPS.
- The results of yield, percentage, and efficiency of grafting, and the data on BP initiator indicated that the grafting reactions of AA and HEMA to starch were successful.
- FTIR analysis confirmed that these were new products of starch and vinyl monomers.
- Most monomers remained in the sample HS-AA-KPS, followed by the sample HS-HEMA-BP, with the fewest in the samples HS-AA-BP and HS-HEMA-AIBN, which confirms a more successful graft polymerization in the presence of these initiators.
- HPLC chromatograms (RID signal) of aqueous extracts of synthesized starch copolymer samples (AA and HEMA) showed that, depending on the type of initiator used, AA BP has the highest peak height and the largest area under the diagram for monomers grafted with the initiator, while HEMA monomer grafted with the AIBIN initiator has the highest peak height and the KPS initiator has the largest surface area.
- The highest uniformity of molar mass was shown in samples of hydrolysed grafted starch, during an elution volume of 2.15–4.0 mL. The smaller molar masses of the grafted samples with an elution volume of 4.2–6.5 mL resulted in poorer homogeneity.
- The viscosity decreased with decreasing starch concentration. All grafted hydrolysed starches increased viscosity compared to non-grafted hydrolysed starches, indicating good stability of the potential starch.
- After impregnation of the yarn in sizing, the yarn mass yarn increased by the amount of the starch applied. Coarser single yarn as well as coarser plied yarn adsorbed or bound more starch, which is associated with a larger volume and more loose structure.
- The sizing process caused an increase in the breaking force. Before grafting, hydrolysed starch had a lower breaking force than grafted starch. The higher starch concentration had a higher breaking force. Starches with HEMA monomer gave better results than AA monomer in terms of breaking force of the yarn, which confirms their more homogenous distribution and good adhesion both on the surface and inside the yarn.
- Sizing reduced elongation at break of yarns and starches with HEMA monomer reduced elongation at break the least.
- Grafted starch proved to be a good agent for abrasion resistance, especially HS-HEMA, which can be ascribed to its good hydrophobicity, thus providing greater surface protection of the yarn. By the hydrolysis of corn starch and subsequent grafting, the yarn hairiness was significantly reduced in all yarns. An increase in the amount of starch caused less hairiness. Higher starch concentration had a better effect, as the number of fibres protruding in the direction of the measuring zones decreased considerably, especially in single yarns.
- Desizing of yarn using ultrasound gave better results than desizing by washing, even at lower size concentrations.
- Optical microscopy of the starch solution showed partially altered granules at different stages of swelling. Longer dormant starch state caused changes on the surface of acid hydrolysed starch granule, the granule surface became less smooth and the size increased.
- Tegewa numbers six and above generally occurred in all grafted starches, meaning that the desizing process was successful.
- On the basis of the analyses carried out, it can be concluded that there is an economic, qualitative, and ecological cost-effectiveness of the return of corn starch to the sizing process, but only by synthesizing and grafting with appropriate initiators, which significantly improves the properties.

## Figures and Tables

**Table 1:** Designations of the obtained copolymers, their meaning and content of the recipe per yarn types.

Copolymer Designation	Meaning of Designations	Graft Schemes
HS	Hydrolysed starch	
HS-AA-AIBN	Hydrolysed starch grafted with acrylamide, initiator azobisisobutyronitrile	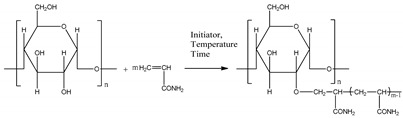
HS-AA-KPS	Hydrolysed starch grafted with acrylamide, initiator potassium persulfate	
HS-AA-BP	Hydrolysed starch grafted with acrylamide, initiator benzoyl peroxide	
HS-HEMA-AIBN	Hydrolysed starch grafted with *2*-*hydroxyethyl methacrylate*, initiator azobisisobutyronitrile	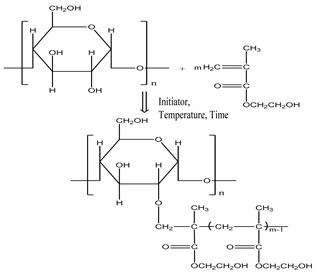
HS-HEMA-KPS	Hydrolysed starch grafted with *2*-*hydroxyethyl methacrylate*, initiator potassium persulfate	
HS-HEMA-BP	Hydrolysed starch grafted with *2*-*hydroxyethyl methacrylate*, initiator benzoyl peroxide	

**Table 2:** Obtained yield values.

Samples		PHS (%)	PriK (%)	PrpK (%)	PEK (%)	KMP (%)
Hydrolysed starch	HS	76.92	-	-	-	-
Grafting of (AA) onto starch	HS-AA-AIBN	-	79.33 ^a^	19.00 ^a^	38.31 ^a^	99.20 ^b^
	HS-AA-KPS	-	80.00 ^a^	20.00 ^a^	40.65 ^a^	98.40 ^b^
	HS-AA-BP	-	81.33	22.00	44.49	98.90
Grafting of HEMA onto starch	HS-HEMA-AIBN	-	84.67	27.00	54.88	98.40
	HS-HEMA-KPS	-	81.33 ^a^	22.00 ^a^	44.81 ^a^	98.20 ^b^
	HS-HEMA-BP	-	83.33 ^a^	25.00 ^a^	50.71 ^a^	98.60 ^b^

where: PHS—yield of starch hydrolysis (%), PriK—yield of grafting (%), PrpK—percentage of grafting (%), PEK—percentage of graft efficacy (%), KMP—conversion of monomer in the polymer (%), a—for significance level 0.05, mean value of the group sample is significantly different from the hypothetical mean value, b—for significance level 0.05, mean value of the group sample is not significantly different from the hypothetical mean value.

**Table 3:** The amount of the retained monomer in the copolymer after grafting.

Samples	Retention Time, min	Concentration of the Residual Monomer in the Copolymer, mg/g
HS-AA-BP	2.505	4.46
HS-AA-AIBN	2.507	4.53
HS-AA-KPS	2.509	6.14
HS-HEMA-BP	2.602	5.75
HS-HEMA-AIBN	2.609	4.46
HS-HEMA-KPS	2.603	5.42

**Table 4:** Individual parameters of chromatograms for copolymer samples.

Samples	Retention Time	Peak Surface Area	Peak Height
HS	5.107	608,958.6	14,139.9
HS-AA-AIBN	5.199	521,071.8	16,214.7
HS-AA-BP	5.296	633,742.5	26,015.8
HS-AA-KPS	5.112	393,332.2	13,030.5
HS-HEMA-AIBN	5.167	522,475.4	15,776.7
HS-HEMA-BP	5.090	532,256.9	14,478.3
HS-HEMA-KPS	5.048	598,744.7	13,734.8

**Table 5:** Different molar masses (g/mol) of HS and copolymers (AA and HEMA).

Samples	Elution Volume 2.15—4.0 mL				Elution Volume 4.2—6.5 mL			
	*Mn*	*Mw*	*Mz*	*D*	*Mn*	*Mw*	*Mz*	*D*
HS	6.68 × 10^5^	1.10 × 10^6^	2.24 × 10^6^	1.65	1.86 × 10^3^	4.32 × 10^3^	7.84 × 10^3^	2.31
HS-AA-AIBN	7.19 × 10^5^	1.54 × 10^6^	3.06 × 10^6^	2.15	2.12 × 10^3^	4.41 × 10^3^	7.90 × 10^3^	2.08
HS-AA-BP	8.45 × 10^5^	1.60 × 10^6^	2.78 × 10^6^	1.89	2.31 × 10^3^	4.63 × 10^3^	7.91 × 10^3^	2.00
HS-AA-KP	7.59 × 10^5^	1.24 × 10^6^	2.32 × 10^6^	1.64	1.99 × 10^3^	4.49 × 10^3^	7.85 × 10^3^	2.26
HS-HEMA-AIBN	8.89 × 10^5^	1.60 × 10^6^	3.34 × 10^6^	1.79	1.98 × 10^3^	4.40 × 10^3^	7.92 × 10^3^	2.22
HS-HEMA-BP	6.81 × 10^5^	1.25 × 10^6^	2.31 × 10^6^	1.83	1.95 × 10^3^	4.43 × 10^3^	7.89 × 10^3^	2.27
HS-HEMA-KP	8.73 × 10^5^	1.20 × 10^6^	2.77 × 10^6^	1.37	2.50 × 10^3^	5.74 × 10^3^	1.15 × 10^4^	2.29

where: *M*n—numerical molecular mass, *M*w—molecular mass, *M*z—molecular mass, D—dispersity (*M*w/*M*n).

**Table 6:** Size viscosity η (mPa·s) at different concentrations; spindle speed 60 rpm, time—15 min to 40 °C; 20 min to 60 °C and 30 min to 85 °C.

Samples		Single Yarn (20 tex and 30 tex)			Plied Yarn (20 × 2 tex and 30 × 2 tex)		
		40 °C	60 °C	85 °C	40 °C	60 °C	85 °C
HS 5/HS-1	η (mPa·s)	38.8	57.1	53.9	11.5	16.3	14.9
	Sd (mPa·s)	0.34	0.82	1.52	1.46	2.21	1.53
	CV (%)	0.88	1.44	2.82	12.69	13.56	10.27
HS-10/HS-3	η (mPa·s)	46.2	65.2	61.9	22.6	37.1	35.5
	Sd (mPa·s)	1.65	1.11	1.19	1.34	1.71	0.96
	CV (%)	3.57	1.70	1.92	5.93	4.61	2.70
HS-15/HS-5	η (mPa·s)	53.6	71.8	70.2	38.8	57.1	53.9
	Sd (mPa·s)	1.31	1.32	1.43	1.18	0.90	1.25
	CV (%)	2.44	1.84	2.04	3.04	1.58	2.32
HS-AA-5/ HS-AA-1	η (mPa·s)	42.9	60.2	58.9	12.9	19.9	16.2
	Sd (mPa·s)	1.54	1.27	1.01	1.70	1.25	1.70
	CV (%)	3.59	2.11	1.71	13.18	6.28	10.49
HS-AA-10/ HS-AA-3	η (mPa·s)	53.9	66.1	63.5	26.6	39.2	37.7
	Sd (mPa·s)	1.65	1.44	1.22	1.57	1.26	1.27
	CV (%)	3.06	2.18	1.92	5.90	3.21	3.37
HS-AA-15/ HS-AA-5	η (mPa·s)	66.5	70.5	68.9	42.9	60.2	58.9
	Sd (mPa·s)	0.82	1.63	1.25	1.25	1.37	1.54
	CV (%)	1.23	2.31	1.81	2.91	2.27	2.61
HS-HEMA-5/HS-HEMA-1	η (mPa·s)	44.8	62.0	60.0	14.3	21.7	20.1
	Sd (mPa·s)	1.70	1.63	1.78	0.92	1.37	1.10
	CV (%)	3.79	2.63	2.97	6.43	6.31	5.47
HS-HEMA-10/HS-HEMA-3	η (mPa·s)	56.8	68.9	64.0	29.7	42.3	42.8
	Sd (mPa·s)	1.53	1.2	1.35	1.31	1.65	1.26
	CV (%)	2.69	1.80	2.11	4.41	3.90	2.94
HS-HEMA-15/HS-HEMA-5	η (mPa·s)	69.3	72.9	70.6	44.8	62.0	60.0
	Sd (mPa·s)	1.20	1.47	1.43	0.92	1.63	1.63
	CV (%)	1.73	2.02	2.02	2.05	2.63	2.72

where: η—size viscosity (mPa·s), Sd—standard deviation (mPa·s), CV—variation coefficient (%).

**Table 7:** Breaking forces and elongation at break of the yarns per yarn counts, sizing monomers and concentrations.

Samples	Count of the Single Yarn								Count of the Plied Yarn							
	20 tex				30 tex				20 × 2 tex				30 ×2 tex			
	F_20_ (cN)	CV (%)	Ɛ_20_ (%)	CV (%)	F_30_ (cN)	CV (%)	Ɛ_30_ (%)	CV (%)	F_20×2_ (cN)	CV (%)	Ɛ_20×2_ (%)	CV (%)	F_20×2_ (cN)	CV (%)	Ɛ_30×2_ (%)	CV (%)
Before sizing	330	8.1	4.1	9.1	459	7.4	5.4	7.1	650	4.4	6.3	6.8	824	5.3	5.1	5.5
HS-5/HS-1	349	9.2	2.9	8.8	478	6.6	3.8	6.5	665	4.8	5.5	7.5	835	4.5	4.6	6.2
HS-10/HS-3	410	8.2	2.8	7.5	546	6.1	3.7	6.3	675	2.8	5.4	8.3	852	4.4	4.5	7.3
HS-15/HS-5	445	7.8	2.7	7.9	574	7.2	3.5	5.5	688	5.2	5.3	9.6	877	3.5	4.3	5.8
HS-AA-5/HS-AA-1	374	7.6	3.1	7.5	486	7.5	4.1	4.7	665	4.6	5.9	8.0	838	6.4	4.7	5.2
HS-AA-10/HS-AA-3	436	7.9	3.0	7.8	555	7.3	4.1	3.9	686	4.1	5.7	7.3	839	5.4	4.5	8.0
HS-AA-15/HS-AA-5	450	7.8	2.9	7.8	586	7.1	3.9	5.2	695	4.6	5.6	5.4	880	8.3	4.4	8.2
HS-HEMA-5/HS-HEMA-1	385	8.5	3.5	8.8	497	8.1	4.5	5.7	671	6.0	6.1	10.2	845	4.7	4.9	10.0
HS-HEMA-10/HS-HEMA-3	443	8.2	3.4	8.1	586	8.1	4.4	4.6	687	4.8	6.0	9.6	860	3.8	4.9	6.5
HS-HEMA-15/HS-HEMA-5	467	7.4	3.4	8.2	601	8.2	4.3	7.3	705	3.8	5.8	6.7	903	5.4	4.7	9.8

where: F_20,30,20×2,30×2_—breaking force according to the yarn designations (cN), Ɛ_20,30,20×2,30×2_—elongation at break according to the yarn designations (%), CV—variation coefficient (%).

**Table 8:** Degree of yarn desizing by using washing/ultrasound.

Samples	Degree of Yarn Desizing; by Using Washing off/ Ultrasound (%)			
	20 tex	30 tex	20 × 2 tex	30 × 2 tex
HS-5/HS-1	80/90	70/78	85/90	75/83
HS-10/HS-3	75/85	66/74	80/85	70/76
HS-15/HS-5	72/82	65/72	77/83	65/70
HS-AA-5/HS-AA-1	92/95	90/95	91/95	90/94
HS-AA-10/HS-AA-3	90/92	89/91	90/93	88/92
HS-AA-15/HS-AA-5	85/88	81/85	86/90	82/86
HS-MK-5/HS-HEMA-1	91/97	93/98	92/97	93/97
HS-MK-10/HS-HEMA-3	90/94	92/95	91/94	92/95
HS-MK-15/HS-HEMA-5	86/88	88/90	85/90	88/92

**Table 9:** Tegewa classification of desizing performance (by classic washing/ultrasound).

Samples	*Tegewa* Number by Using Washing/ultrasound			
	20 tex	30 tex	20 × 2 tex	30 × 2 tex
HS-5/HS-1	6/6.5	5/7	6.5/7	6/7.5
HS-10/HS-3	5.5/6	5/6.5	6/6	6/7
HS-15/HS-5	5/6.5	4.5/6.5	6/7	5,5/7
HS-AA-5/HS-AA-1	7.5/8	7/8	7/7	6.5/7.5
HS-AA-10/HS-AA-3	7.5/8	6.5/8	7/8	6.5/8
HS-AA-15/HS-AA-5	6/6.5	6/7.5	6/7	6/8
HS-MK-5/HS-HEMA-1	8/8	8/8	7.5/8	7/8
HS-MK-10/HS-HEMA-3	7.5/7.5	8/8	7.5/7.5	7/7.5
HS-MK-15/HS-HEMA-5	7/7.5	7.5/8	7/7	7/7.5
